# An Unusual Case of Non-typhoidal Salmonella Bacteremia Causing Life-Threatening Aortitis

**DOI:** 10.7759/cureus.54645

**Published:** 2024-02-21

**Authors:** Fawwad A Ansari, Stefan Gafoor, Mubashira Aftab, Zola Nlandu

**Affiliations:** 1 Internal Medicine, Piedmont Athens Regional Medical Center, Athens, USA; 2 Internal Medicine, Fazaia Medical College, Islamabad, PAK

**Keywords:** bacteremia, gram-negative bacteremia, salmonella infection, salmonella enterica, aortic ulcer, salmonella aortitis, mycotic aortic aneurysm

## Abstract

Non-typhoidal *Salmonella* typically presents with gastroenteritis. However, an invasive *Salmonella* infection, which may be typically seen in immunocompromised patients, has a propensity for aortic involvement, especially in patients with risk factors for atherosclerosis. Here we present a 60-year-old female with multiple comorbid conditions and currently on immunosuppressants for rheumatoid arthritis, who presented with nausea, vomiting, and fever of three weeks duration and was found to have *Salmonella* bacteremia. Blood cultures were positive for *Salmonella enterica*. Computed tomography (CT) abdomen with contrast was concerning for mycotic aortitis. The patient underwent endovascular repair of an aortic ulcer and was treated with a six-week course of ceftriaxone.

Mycotic aneurysm is a rare but potentially fatal complication of invasive *Salmonella* infection. It occurs typically in older men with atherosclerotic risk factors. It mostly presents as fever, back pain, and/or abdominal pain. Our patient was a middle-aged female who presented with non-specific symptoms. CT angiogram is the diagnostic modality of choice and treatment may require surgical vascular repair and long-term antibiotics. A high level of suspicion is needed to diagnose *Salmonella-related* mycotic aneurysm/aortitis. Early diagnosis and treatment may improve the mortality.

## Introduction

*Salmonella* infections are divided into five subgroups: chronic carrier state, bacteremia, gastroenteritis, localized infection, and enteric fever.

Most non-typhoidal *Salmonella* infections typically present with gastroenteritis and fever, resolving without serious complications. However, some serovars may cause systemic disease, especially in immunocompromised patients [1]. One of the most severe complications of extraintestinal or invasive non-typhoidal *Salmonella* infection is mycotic aneurysms [2]. Interestingly, *Salmonella enterica*, serovar Enteritidis, is the second most common cause of all bacterial mycotic aneurysms worldwide, second only to *Staphylococcus aureus* [2-4]. The incidence of rupture is higher than that of the atherosclerotic aneurysm and is associated with a higher rate of mortality. Early diagnosis and adequate treatment are significant for survival [4]. The objective of this case report is to present a patient with *Salmonella* enteritis complicated by bacteremia and mycotic aneurysm, highlighting diagnostic and treatment pearls.

## Case presentation

A 60-year-old female with a past medical history of type 2 diabetes mellitus, hypertension, and rheumatoid arthritis currently being managed with methotrexate and rituximab, presented due to persistent nausea, vomiting, and diarrhea for three weeks. She reported a history of dining at a restaurant three weeks prior to her presentation, after which she developed these symptoms. The patient reported having two to three episodes of vomiting and loose stools daily since that time. It was associated with intermittent abdominal pain localized to the epigastric and umbilical region, with no radiation, decreased appetite, subjective fevers, and malaise. She was unaware of any sick contacts and reported no recent travel.

On presentation, she had a temperature of 100° F (Fahrenheit) and was otherwise hemodynamically stable. On exam, she was ill appearing with rigors. Her abdomen was neither distended nor tender. The remainder of her physical exam was without significant findings.

On initial lab workup (Table 1), her complete metabolic panel (CMP) revealed an elevated aspartate aminotransferase (AST) 57 IU/L, alanine aminotransferase (ALT) 48 IU/L, total bilirubin 1.8 mg/dL, and alkaline phosphatase (ALP) 133 IU/L. Her complete blood count (CBC) showed a hemoglobin of 11.9 g/dl and leukocytosis with a white blood cell (WBC) count of 13,400/mm^3^. The human immunodeficiency virus (HIV) screen was non-reactive. Blood cultures drawn on admission were positive for *Salmonella enterica*, and the susceptibility report demonstrated pan-susceptibility. The patient was commenced on intravenous ceftriaxone 2 grams daily.

**Table 1:** Laboratory workup for the patient ALT: alanine aminotransferase; AST: aspartate aminotransferase; ALP: alkaline phosphatase; HIV: human immunodeficiency virus; mm^3^: cubic millimeters; g/dL: grams per deciliter; mg/L: milligrams per deciliter; IU/L: international units per liter

Variable	Labs (on admission)	Reference range
White blood cells (cells/mm^3^)	13,400	4.5 to 11.0
Hemoglobin (g/dL)	11.9	12.0 to 16.0
ALT (IU/L)	48	10.0 to 40.0
AST (IU/L)	57	10.0 to 40.0
ALP (IU/L)	133	30.0 to 120.0
Total bilirubin (mg/dL)	1.8	0.3 to 1.0
HIV screen	Non-reactive	N/A
Blood culture	Salmonella enterica	N/A

**Figure 1:**
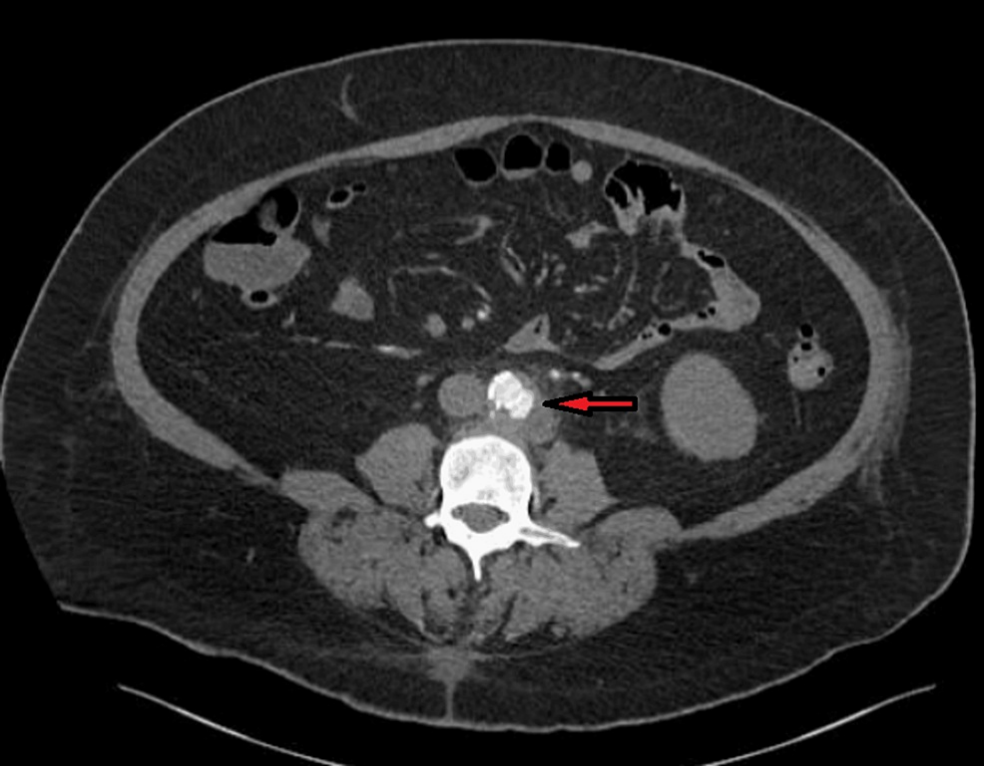
Axial view of CT angiogram of the abdomen Axial view of the CT angiogram of the abdomen demonstrating inflammatory stranding just cranial to the aortic bifurcation with an associated ulcer (arrow).

A computed tomography (CT) scan of her abdomen and pelvis with contrast (Figure 1) was obtained due to the acuity of her symptoms, subsequently revealing circumferential periaortic fat stranding, just proximal to the aortic bifurcation with concern for a penetrating aortic ulcer and raising suspicion for mycotic aortitis. There were no signs of rupture.

**Figure 2:**
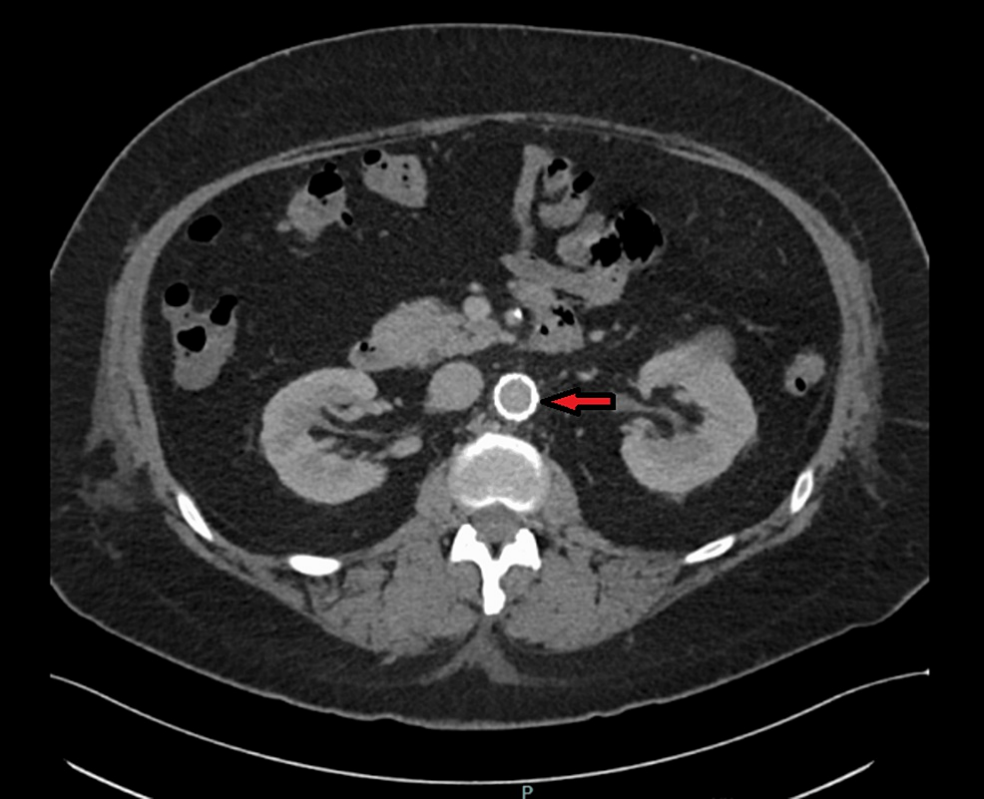
Axial view of CT angiogram of the abdomen post-endovascular repair Axial view of the repeat CT angiogram of the abdomen post-endovascular aortic repair, demonstrating placement of aorto-bi-iliac stent, with similar inflammatory stranding in the region of previously noted penetrating ulcer (arrow).

In view of a possible penetrating ulcer, the vascular surgery team was consulted. The following day, the patient underwent endovascular repair of the aorta (Figure 2). The procedure was without any complications.

Repeat blood cultures done on day two and day ten from her initial presentation were without any bacterial growth. She was subsequently discharged, to complete a six-week course of IV ceftriaxone 2 grams once daily, with appropriate outpatient follow-up.

## Discussion

*Salmonella* species are motile gram-negative facultative anaerobic bacilli of the family Enterobacteriaceae. Non-typhoidal *Salmonella* species are widely disseminated in nature and are usually associated with some animals like chickens and turtles [5]. Gastroenteritis is the most common clinical presentation of non-typhoidal *Salmonella* infection. Approximately 5% of people develop bacteremia or focal invasive infections such as meningitis, osteomyelitis, endovascular infection, or septic arthritis [6].

Mycotic aneurysms are localized abnormal dilatations of the arterial walls that develop secondary to an infective process that causes destruction of the vessel wall. They are rare and represent only 2.6% of all abdominal aortic aneurysms [7]. Most patients with aortitis due to *Salmonella* have pre-existing atherosclerotic disease at the site of the subsequently infected aneurysm [8]. In one study of patients with bacteremia due to *Salmonella*, 25% of those 50 years of age developed an endothelial infection [9]. The clinical picture of aortitis due to *Salmonella* consists almost always of fever, back pain, and/or abdominal pain [10].

The rates of invasive infections and death are generally higher among infants, older adults, and people with immunosuppressive conditions, hemoglobinopathies, and malignant neoplasms [6]. Bacteremia due to *Salmonella* has been noted in many patients living with HIV infection. However, aortitis rarely occurs in these patients likely because they are usually younger and without the atherosclerotic risk factors [11]. Mycotic aneurysm or aortitis is a rare but potentially fatal complication of invasive *Salmonella* infection. 

Our patient was a middle-aged female living with diabetes, hypertension, as well as rheumatoid arthritis being treated with methotrexate and rituximab. We believe her immunocompromised state was a contributory factor for her invasive disease and her other comorbid conditions, such as diabetes mellitus and hypertension (atherosclerotic risk factors), likely played a role in mycotic aneurysm development.

Also, our case highlights a low threshold of suspicion is required to diagnose mycotic aneurysm or aortitis in patients with *Salmonella* bacteremia. Commonly mycotic aneurysm or aortitis manifests as fever, chills, along with pain at the location of an aneurysm, usually the back or the abdomen [1,4]. However, in our patient, the only symptoms were persistent fever, chills, nausea, vomiting and diarrhea. At the time of presentation, she did not report any abdominal or back pain, except occasional abdominal cramps, which could be mistaken for *Salmonella* gastroenteritis.

CT angiography remains the diagnostic tool of choice with sensitivity greater than 95% [2]. Management includes a multidisciplinary approach with surgical repair of aneurysms and long-term antibiotics. Two surgical approaches are recommended: open aneurysm repair or endovascular aneurysm repair. Open repair has higher mortality but better infectious source control. The overall outcomes between the two approaches, however, appear to be comparable and the procedure is chosen based on the aneurysm location, the extent of the infection, the fitness of the patient, and the surgeon's preference [12]. In patients treated with surgery, the two-year survival is about 20% and death usually results from graft reinfection and graft leakage [13]. Usually, a six-week course of intravenous antibiotics is recommended but some patients may require a longer duration of antibiotics. Evidence has shown poor outcomes and a high rate of recurrence with treatment with antibiotics alone [2]. For our patient, similarly, based on the recommendations, we managed her with endovascular aortic repair and long-term antibiotics, with close outpatient follow-up.

## Conclusions

Non-typhoidal *Salmonella* aortitis is a life-threatening condition. Due to the vague symptoms that patients may present with, there should be high clinical suspicion in the right clinical context, especially in immunocompromised patients or those with significant risk factors for atherosclerosis. Antibiotic therapy and surgical intervention are the mainstay of treatment. Our patient was treated with a prolonged course of intravenous antibiotic therapy and aortic endovascular repair to good effect.
